# Characterization of Japanese Encephalitis Virus Isolated from Persistently Infected Mouse Embryo Cells

**DOI:** 10.3390/tropicalmed9050117

**Published:** 2024-05-16

**Authors:** Yume Kondo, Tomoyoshi Komiya

**Affiliations:** Faculty of Health and Medical Sciences, Hokuriku University, 1-1, Taiyogaoka, Kanazawa 920-1180, Ishikawa, Japan

**Keywords:** Japanese encephalitis virus, persistent cell infection, E protein genotype

## Abstract

Japanese encephalitis virus (JEV) has a positive-sense single-stranded RNA genome and belongs to the genus *Flavivirus* of the family *Flaviviridae*. Persistent JEV infection was previously shown in pig blood cells, which act as a natural reservoir of this virus. We aimed to determine the pathogenicity factors involved in persistent JEV infection by analyzing the pathogenicity and genome sequences of a virus isolated from a persistent infection model. We established persistent JEV infections in cells by inoculating mouse fetus primary cell cultures with the Beijing-1 strain of JEV and then performing repeated infected cell passages, harvesting viruses after each passage while monitoring the plaque size over 100 generations. The virus growth rate was compared among Vero, C6/36, and Neuro-2a cells. The pathogenicity was examined in female ICR mice at several ages. Additionally, we determined the whole-genome sequences. The 134th Beijing-1-derived persistent virus (ME134) grew in Vero cells at a similar rate to the parent strain but did not grow well in C6/36 or Neuro-2a cells. No differences were observed in pathogenicity after intracerebral inoculation in mice of different ages, but the survival time was extended in older mice. Mutations in the persistent virus genomes were found across all regions but were mainly focused in the NS3, NS4b, and 3′NCR regions, with a 34-base-pair deletion found in the variable region. The short deletion in the 3′NCR region appeared to be responsible for the reduced pathogenicity and growth efficiency.

## 1. Introduction

Japanese encephalitis (JE) is an acute inflammatory disease of the central nervous system caused by infection with the JE virus (JEV) [1], which is closely related to the West Nile virus, dengue virus, St. Louis encephalitis virus, and Murray Valley encephalitis virus [2]. This flavivirus is a small, enveloped virus containing a single-stranded positive-sense RNA genome of approximately 11,000 nucleotides. The genome has a single open reading frame, which encodes three structural proteins (capsid, premembrane or membrane, and envelope [E]) and seven nonstructural proteins (NS1, NS2a, NS2b, NS3, NS4a, NS4b, and NS5) between the 5’ nontranslated region (NTR) and the 3′NTR [2]. The viral E protein, which is modified by glycosylation and dimerization during virion assembly, serves as the cell–receptor binding protein and the fusion protein for viral attachment and entry into the host. The E protein is directly associated with neutralization [3,4].

JEV is the leading cause of viral encephalitis in Southeast Asia, India, and China; the geographical distribution of JEV is also expanding, most recently into southwest India, the eastern Indonesian archipelago, New Guinea, and the Torres Strait of northern Australia [5]. Annually, ~35,000 human cases of JE are reported, resulting in ~10,000 deaths and a high incidence of neuropsychiatric deficits among survivors. Children and non-immune adults are predominantly at risk of contracting the disease in endemic areas. JEV is an arbovirus and is transmitted by Culex mosquitoes to its vertebrate hosts (including wild and domestic birds and pigs); human infections with JEV do not result in a sufficiently high viremia for maintenance of the transmission cycle. JE is also a veterinary disease with occasional fatal outcomes in horses, as well as abortions and abnormal births in pigs [6].

Chronic disease associated with persistent RNA virus infections represents a key public health concern. While human immunodeficiency virus-1 and hepatitis C virus are perhaps the most well-known examples of persistent RNA viruses that cause chronic disease, evidence suggests that many other RNA viruses, including re-emerging viruses, such as chikungunya virus, Ebola virus, and Zika virus, establish persistent infections [7,8,9]. JEV has been reported to cause persistent infection in porcine blood cells [10]. However, the mechanisms by which RNA viruses drive chronic disease are poorly understood. In this study, a persistent JEV strain was established and characterized based on virus growth, viral pathogenesis, and viral sequencing analyses.

## 2. Materials and Methods

### 2.1. Animals

All studies were performed following the guidelines specified by the Animal Experimentation and Ethics Committee of the Kitasato Institute Research Center for Biologicals (protocol number R-2016-028). Pregnant and 4-week-old ICR female mice were obtained from Japan SLC Inc. (Hamamatsu, Japan). The animals were maintained under specific-pathogen-free conditions.

### 2.2. Cells and Viruses

Vero (JCRB9013), Neuro-2a, and SK-N-SH cells were obtained from the Japanese Collection of Research Bioresources Cell Bank (National Institutes of Biomedical Innovation, Health, and Nutrition, Osaka, Japan), and C6/36 cells were obtained from Prof. Koichi Morita at Nagasaki University. The Vero, Neuro-2a, and SK-N-SH cells were maintained in minimum essential medium (MEM, Thermo Fisher Scientific, Waltham, MA, USA) supplemented with 10% fetal bovine serum (FBS), and the C6/36 cells were maintained in MEM supplemented with 10% FBS and MEM non-essential amino acids (NEAA, Thermo Fisher Scientific, Waltham, MA, USA). The JEV Beijing-1 strain was added to 10% suckling mouse brain homogenates in Hanks’ balanced salt solution containing 20 mM HEPES buffer (pH 8.0) and 02% bovine serum albumin.

### 2.3. Established Persistent Virus Strain

Mouse embryo cells were propagated from the age of 16–18 days and were maintained in MEM supplemented with 10% FBS. The mouse embryo cells were grown at 37 °C and inoculated with JEV Beijing-1 virus at a multiplicity of infection (MOI) equal to 10. After incubation at 37 °C for several days, the cells were subcultured every 2–4 days until more than 100 passages had been performed, while checking the virus titer and virus plaque size using a plaque formation assay on Vero cells, as previously described [11]. Then, after more than 100 passages, the ME134 passage virus was used based on its plaque size index, and the viruses were purified by means of plaque picking three times; the ME134 strain was thus established as the persistent virus. In the past, our laboratory has produced persistently infected cells for the purpose of producing attenuated strains for the development of live vaccines.

### 2.4. Immunofluorescence Assay for JEV-NS1 Protein

ME cells were subcultured in eight-well chamber slides (Thermo Fisher Scientific, Waltham, MA, USA) at 37 °C and 5% CO_2_ for one day and fixed using methanol. The viral NS1 protein was visualized using an FITC-labeled anti-JEV NS1 antibody, and nuclear staining was achieved using DAPI.

### 2.5. Virus Growth Rate

Vero cells, Neuro-2a cells, C6/36 cells, and SK-N-SH cells were cultured in a T-75 flask, and the cells were infected with the persistent virus at MOI = 0.01. After adsorption for 90 min, the cells were washed in PBS and maintained in MEM with 2% FBS and NEAA (only added for C6/36 cells) at 37 °C and 5% CO_2_ for 6 days.

### 2.6. Challenge Test in ICR Mice

Herein, 2–3-day-old ICR mice and 4-week-old ICR mice (n = 5/group) were inoculated with 10^4^ PFU virus via the intracerebral route and observed for 16 days. As a mock-infected control, PBS was inoculated in each study.

### 2.7. Sequence Analysis

RNA was extracted from virus-infected C6/36 cells using the RNeasy Mini Kit (Qiagen, Tokyo, Japan), reverse-transcribed using AMV reverse transcriptase with a random primer (Takara, Tokyo, Japan), and then used for polymerase chain reaction (PCR) with LA Taq DNA polymerase (Takara). PCR products were purified with the QIAquick PCR Purification Kit (Qiagen) and used for direct sequencing with the BigDye Terminator Cycle Sequencing Kit (Applied Biosystems, Foster City, CA, USA). To determine the sequence of the 5′ genome end, the 5′ RACE system (Invitrogen, Carlsbad, CA, USA) was used. For the 3′ end, the RACE system was applied to the anti-sense genome.

## 3. Results

### 3.1. Established Persistent Virus Strain

**Figure 1:**
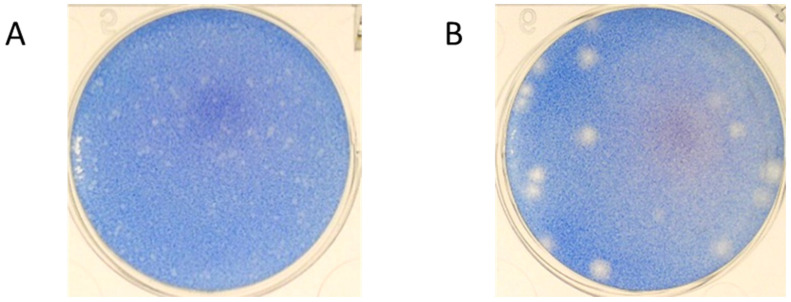
Plaque sizes of the persistent and parent viruses. The ME134 persistent strain virus (**A**) and the Beijing-1 strain virus (**B**) were titrated on Vero cells.

**Figure 2:**
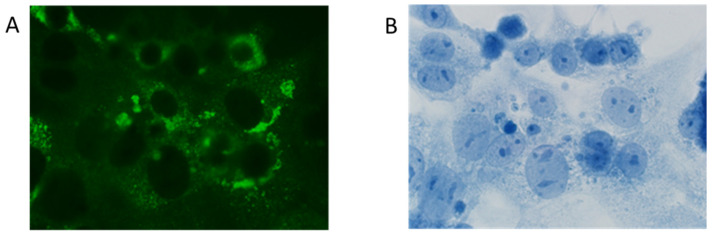
Immunofluorescence assay of ME cells using anti-JEV NS1 antibody after one day of subculture. NS1 protein (**A**) and nuclei (**B**) were detected using the anti-JEV NS1 antibody and DAPI, respectively, in mouse embryo cells infected with ME virus.

A persistent virus strain was established after 100 passages of inoculation at MOI = 10 in mouse embryo cells with a selection marker for a small plaque size. The ME134 virus was established from the JEV Beijing-1 strain. Each plaque size was small compared with that of the parent virus (Figure 1), and the virus growth titers were 10^2^ to 10^5^ PFU/mL, without any cytopathic effect (CPE). The immunofluorescence assay against the JEV-NS1 protein revealed that almost all subcultured cells inoculated with the persistent ME virus (ME cells) were infected (Figure 2).

### 3.2. Virus Growth Rate

**Figure 3:**
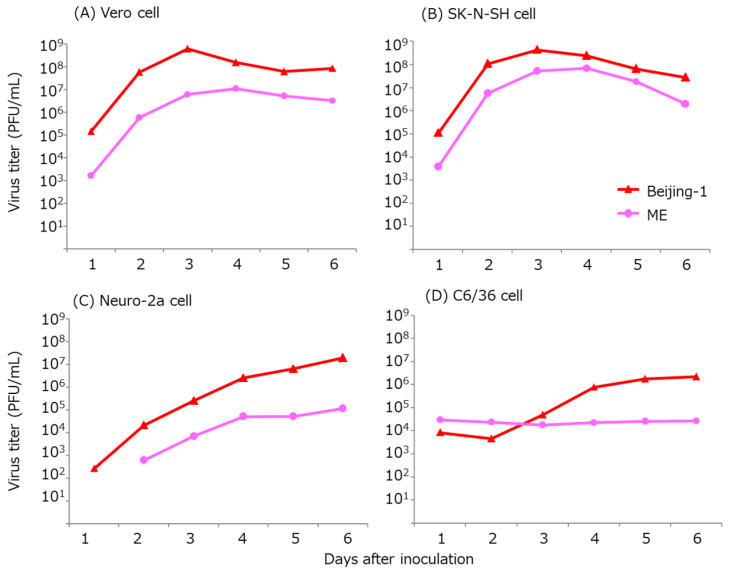
Virus growth rates for the persistent and parent virus strains. Vero cells (**A**), SK-N-SH cells (**B**), Neuro-2a cells (**C**), and C6/36 cells (**D**) were infected with the viruses at MOI = 0.01 and cultured for 6 days.

The Vero, Neuro-2a, C6/36, and SK-N-SH cells were infected with the persistent virus and the parent virus, and the growth curves and INF production were compared. The cells were inoculated at MOI = 0.01 and cultured for 6 days at 37 °C and 5% CO_2_. CPE was observed in the cells, except for in C6/36 cells infected with the parent viruses, but morphological changes were not observed in the cells infected with the persistent virus. The ME134 persistent virus showed a lower virus growth rate and a lower peak virus titer (Figure 3).

### 3.3. Mouse Survival Rate

**Figure 4:**
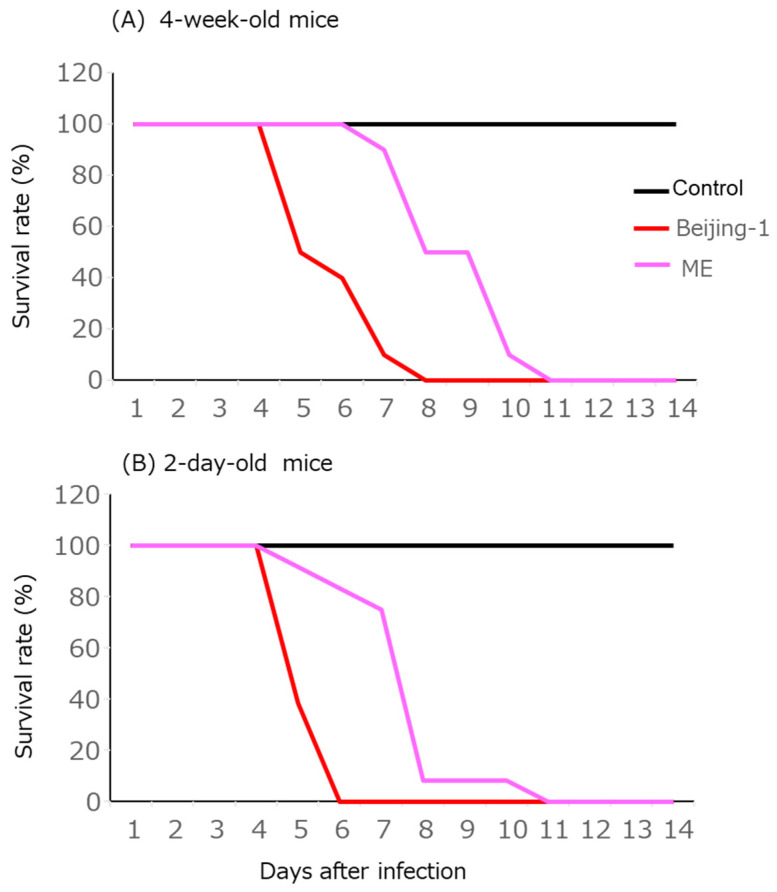
Survival rate after infection with 10^4^ or 10^5^ of each virus in 4-week-old ICR mice (**A**) and 2-day-old ICR mice (**B**), respectively, inoculated via the i.c. route.

In the experiments, 2–3-day-old and 4-week-old female ICR mice were intracranially inoculated with the persistent and parent viruses. Mice inoculated with the parent virus died faster than did those inoculated with the persistent virus. Lethality was delayed for several days in both age groups (Figure 4).

### 3.4. Sequence Analysis

**Figure 5:**
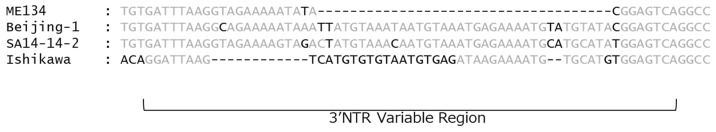
Sequence analysis in the 3′ noncoding region. The SA-14-14-2 strain is a live attenuated vaccine strain, and the Ishikawa strain is a genotype I strain isolated in Japan.

The RNA genomes of the persistent and parent viruses were analyzed, and all amino acid sequences were compared. The amino acid mutations that occurred in the persistent virus were clustered in the NS3 and NS4B proteins, and each amino acid mutation occurred in both genomes; no region-specific mutations were observed (Table 1). Moreover, the ME134 genome had a 34 bp deletion in the 3′NTR variable region (Figure 5).

## 4. Discussion

In vivo and virus studies to establish persistent infections using flaviviruses have been reported for yellow fever [12], Murray Valley encephalitis virus [13], and JEV [14]. However, it is difficult to find stable cell lines for studies of persistent infection. Pathogenicity and whole-genome sequencing and characterization were carried out using a virus strain produced in persistently JEV-infected cells, previously established in fetal mouse cells over 134 passages for the purpose of developing live vaccine strains.

The lethality of ME134, a persistent virus derived from the JEV Beijing-1 strain, was delayed compared with that of the parental strain in both 2–3-day-old suckling mice and 4-week-old mice, indicating a decrease in virulence or viral proliferation due to persistent infection. JEV can infect and multiply widely in hosts ranging from insects to humans and is not host-specific. In addition, it can infect a wide range of cultured cell lines, and it is not known whether there are viral receptors that determine the host range. Therefore, growth experiments were conducted in several cell types, including mammalian, neuronal, and mosquito cell-derived lines. Cell proliferation tests also showed a decrease in viral proliferation in Vero, C6/36, and Neuro-2a cells regardless of their origin, indicating the same trend as in the mouse experiments. This result was similar to that observed for a virus produced in persistently infected cells, developed using the strain of cells reported by Guohe et al. [14].

The genetic analysis showed that the ME134 strain had many amino acid mutations from the parental strain, suggesting differences in its virulence and proliferation. The NS3 region encodes a protein involved in viral RNA replication [15], and the NS4B region encodes a protein involved in apoptosis and encephalitis [16]. The fact that mutations were found in the entire region is thought to be a useful clue to the function of the NS region, which is still unknown. ME134 showed a large genetic defect in the 3′NTR region, which is involved in viral RNA replication, suggesting that the reduction in viral RNA replication and the regulation of cell apoptosis may be responsible for persistent infection. The genetic analysis of the parental strain and the attenuated mutant strain identified the E protein as a neurotoxicity-related region on the viral side. An analysis of the attenuated mutant strain SA14-14-2, which is a live vaccine, showed that amino acid 279 of the E protein was mutated from Met to Lys in the attenuated mutant strain, and this hinge region is considered to be involved in neurotoxicity [17]. On the other hand, Yasui et al. also found that amino acid 138 of the E protein is involved in toxicity based on a genetic analysis of attenuated strains [18]. In the present results, no amino acid mutations in the two E protein regions were observed, suggesting that other regions are involved in virulence against mice.

Recently, JEV genotype III has been replaced by type I in terms of prevalence in Japan, which may be related to the decrease in the number of JEV patients. The characteristic mutation among the genotypes is a gene deletion in the 3′NTR region [19], which is consistent with our analysis of the characteristics of viruses produced by persistently infected cells, and we believe that some form of persistent viral infection may occur in nature [20]. The in vitro cell proliferation and genetic analysis results from this study are concordant with this conclusion.

Meanwhile, we have recently focused our attention on wild boars as amplifiers of JEV, similar to pigs, and are conducting epidemiological studies based on the idea that the virus may be persistently transmitted during overwintering within individual boars [21,22,23]. The detection of IgM antibodies, which indicate an initial JEV infection, in wild boars captured during the winter suggests that persistent infection in nature may play a larger role in JEV ecology than the present experimental results suggest, and further clarification of the host–host interrelationships is needed.

## 5. Conclusions

Our findings show that the pathogenicity of the ME134 virus in mice was considerably reduced compared with that of the parent strain. The short deletion in the 3′NCR region appears to be responsible for the reduced pathogenicity and growth efficiency, as seen in recent genotype I isolates and several other reports in Japan.

## Figures and Tables

**Table 1:** Complete sequence and amino acids of each virus.

	DNA Position	AA Position	Beijing-1	ME134
Capsid	97	1	M	T
	246	51	I	
	402	102	N	
	456	121	V	
prM	516	141	I	
	606	171	M	
M	769	225	T	I
	814	240	D	G
	861	256	I	
	974	300	I	
E	995	307	M	I
	1128	345	S	
	1215	374	A	P
	1224	377	E	
	1346	417 = E123	R	R
	1389	432 = E138	E	
	1452	453	V	
	1504	470	I	
	1512	473	K	
	1804	570	S	
	1846	584	K	
	1893	600	E	G
	2166	691	Y	F
	2230	712	A	
	2394	767	I	
NS1	2499	802	I	
	2509	805	R	
	2689	865	V	
	2775	894	A	S
	3369	1092	I	
	3523	1143	R	
	3530	1145	D	E
NS2A	3610	1172	K	R
	3661	1189	V	
	3850	1252	A	
	3873	1260	F	
	3886	1264	S/L	L
	3981	1296	V	
	4069	1325	I	
	4098	1335	A	
NS2B	4233	1380	F	V
	4468	1458	F	Y
NS3	4784	1563	M	I
	5032	1646	F	
	5152	1686	T	
	5736	1881	Q	K
	6151	2019	G	
	6159	2022	F	
NS4B	6970	2292	K	T
	7167	2358	V	M
	7450	2452	G	
	7561	2489	V	A
	7660	2522	K	R
NS5	7744	2550	E	
	8499	2802	D	
	8617	2841	K	R
	10,257	3388	R	
	10,317	3408	K	E

AA = amino acids; Beijing-1 = parent strain; ME-134 = persistent strain.

## Data Availability

The data presented in this study are available upon request from the corresponding author.
